# Supplementary material for: Cortical tracking of speech in noise accounts for reading strategies in children
Source: PLoS Biol. 2020 Aug 26;18(8):e3000840. doi: 10.1371/journal.pbio.3000840 (PMC7478533; doi:10.1371/journal.pbio.3000840)
Supplement: S1 Methods — (DOCX) [file pbio.3000840.s001.docx]

# Supporting Information

## S1 Methods: Assessment of the degree of energetic masking

We characterized the degree of energetic masking introduced by the different types of noise, that is their degree of spectrotemporal overlap with the attended speech.

As a first step, we computed the spectrogram of the attended speech and that of the noise for each type of noise in each of the 4 versions of each of the 12 videos. Spectrograms were derived using 50-ms-long hanning windows sliding by steps of 25 ms, and with 5-Hz frequency resolution in the range 100–7000 Hz. The modulus of the Fourier coefficients were converted to a dB scale (log-transformed and multiplied by 20), which we denote *S*_attended_(*t*,*f*) for the attended speech and *S*_noise_(*t*,*f*) for the noise, with *t* indexing time and *f* indexing frequency. Figure S1 presents the spectrogram of a 4-s excerpt of attended speech and all noises.

Indices of energetic masking ranging from 0 (no masking) to 1 (substantial masking) were derived from spectrograms. For that, we identified the indices of the 10% highest values of *S*_attended_(*t*,*f*), i.e., the set *N* so that *S*_attended_(*t_n_*,*f_n_*) > *S*_attended_(*t_x_*,*f_x_*) for all indices nNand xN. For each index nN, we estimated the local energetic masking index as *M*(*n*) = 1 – (*S*_attended_(*t_n_*,*f_n_*)–*S*_noise_(*t_n_*,*f_n_*))/(10 dB) further thresholded between 0 and 1. Accordingly, *M*(*n*) = 0 when noise power (*S*_noise_(*t_n_*,*f_n_*)) is at least 10 dB lower than attended speech power (*S*_noise_(*t_n_*,*f_n_*)), *M*(*n*) = 1 when noise power exceeds attended speech power, and e.g., *M*(*n*) = 0.6 when noise power is 4 dB below speech power. The global energetic masking index was simply taken as the mean of *M*(*n*) across all nN. The global energetic masking indices for each type of noise (12 videos times 4 versions) were averaged, and compared between types of noise with independent sample *t*-tests.

This showed that the global energetic masking was lowest for the least-energetic non-speech noise (0.00 ± 0.00; *ps* < 0.0001 for the comparison with the 3 other noises), highest for the most-energetic non-speech noise (0.428 ± 0.035; *ps* < 0.0001), and similar among opposite- and same-gender babble noises (0.310 ± 0.035 and 0.312 ± 0.035; *t*(94) = 0.28, *p* = 0.78).
