# Supplementary material for: Cortical tracking of speech in noise accounts for reading strategies in children
Source: PLoS Biol. 2020 Aug 26;18(8):e3000840. doi: 10.1371/journal.pbio.3000840 (PMC7478533; doi:10.1371/journal.pbio.3000840)
Supplement: S2 Methods — (DOCX) [file pbio.3000840.s002.docx]

# Supporting Information

## S2 Methods: Preprocessing of brain and behavioral indices

All behavioral and nCTS measures were corrected for IQ, age, and time spent at elementary school, and for outliers. For simplicity, we refer to the standardized IQ, age, and time spent at elementary school as the regressors.

An iterative procedure was used to simultaneously control for regressors and fix outliers. First regressors were regressed out of each measure with an amount of regularization equal to 0.1% the maximal eigenvalue of the regressors’ covariance matrix. Then measures deviating by more than 3 standard deviations from the mean were removed from the distribution. This procedure was repeated until there were no more outliers. Discarded data points were then set to the mean plus or minus 3 standard deviations.
