# Supplementary material for: Cortical tracking of speech in noise accounts for reading strategies in children
Source: PLoS Biol. 2020 Aug 26;18(8):e3000840. doi: 10.1371/journal.pbio.3000840 (PMC7478533; doi:10.1371/journal.pbio.3000840)
Supplement: S3 Methods — nCTS, normalized cortical tracking of speech. (DOCX) [file pbio.3000840.s003.docx]

# Supporting Information

## S3 Methods: Extraction of the relevant features of nCTS

In total, we derived 8 features of nCTS in SiN conditions based on the significant effects of hemisphere and conditions highlighted in Table 2 and Figure 2. There were 4 features for phrasal nCTS: (i) the mean nCTS (the mean standardized nCTS across conditions), (ii) the informational modulation in nCTS (the difference in standardized nCTS between babble and non-speech noise conditions averaged across all other factors), (iii) the visual modulation in nCTS (the difference in standardized nCTS between lips and pics visual conditions averaged across all babble noise conditions), and (iv) the hemispheric difference in nCTS (the contrast in standardized nCTS between left and right hemispheres averaged across all babble noise conditions). The same 4 features were used for syllabic nCTS except that visual and hemispheric modulations were evaluated based on averages across all other factors (and not just across babble noise conditions since visual and hemispheric modulations were seen in all noise conditions for syllabic nCTS).
