# Supplementary material for: Cortical tracking of speech in noise accounts for reading strategies in children
Source: PLoS Biol. 2020 Aug 26;18(8):e3000840. doi: 10.1371/journal.pbio.3000840 (PMC7478533; doi:10.1371/journal.pbio.3000840)
Supplement: S4 Methods — (DOCX) [file pbio.3000840.s004.docx]

# Supporting Information

## S4 Methods: Partial information decomposition

As in previous applied studies [1,2], we measure redundancy with pointwise common change in surprisal for Gaussian variables [3]. Continuous data values were first subjected to a rank-normalisation (copula-normalised) [4] before being treated as Gaussian variables. A crucial advantage of this redundancy measure as opposed to other PID implementations is that it measures the overlapping information content at the pointwise level and therefore can be interpreted as a within sample (here participant) measure of redundant prediction, directly linked to the coding interpretations of MI. An advantage of the PID over variance partitioning approaches is that unique variance explained might, like conditional mutual information, be confounded by synergistic effects [5], whereas PID with common change in surprisal gives the true unique contribution. While the PID has so far been applied within subject to trial data, information theoretic quantities can also be applied as a second level analysis, where each participant is a sample [6].

The statistical significance of the different information values was assessed with a nonparametric permutation test [7]. A permutation distribution (based on 10,000 different random permutations) was computed for each information value by randomly permuting children’s target values (i.e., assigning target values of child 1, 2, 3, …, 73 to, e.g., child 34, 20, 61, …, 19). This permutation scheme specifically disrupts the associations between explanatory and target variables while preserving the associations within all explanatory variables and within all targets variables. For each information value, a significance level was computed as the proportion of values from the permutation distribution exceeding the observed value. We also computed the mean and standard deviation of the permutation distribution values to convert information measures into z-scores. Note that, owing to the generality of permutation statistics, significance levels are exact regardless of the strengths and weaknesses of the method used to estimate associations (here PID); and owing to the specificities of the permutation scheme we used, significance levels intrinsically take into account the dimensionality of the explanatory and target sets. Still, for cross-checking, some information values were also compared to their distribution obtained for explanatory variables permuted across subjects.

## References

1. Park H, Ince RAA, Schyns PG, Thut G, Gross J. Representational interactions during audiovisual speech entrainment: Redundancy in left posterior superior temporal gyrus and synergy in left motor cortex. PLoS Biol. 2018;16: e2006558. doi:10.1371/journal.pbio.2006558
2. Daube C, Ince RAA, Gross J. Simple Acoustic Features Can Explain Phoneme-Based Predictions of Cortical Responses to Speech. Curr Biol. 2019;29: 1924–1937.e9. doi:10.1016/j.cub.2019.04.067
3. Ince R. Measuring Multivariate Redundant Information with Pointwise Common Change in Surprisal. Entropy. 2017. p. 318. doi:10.3390/e19070318
4. Ince R. Measuring Multivariate Redundant Information with Pointwise Common Change in Surprisal. Entropy. 2017. p. 318. doi:10.3390/e19070318
5. Daube C, Giordano B, Schyns P, Ince R. Quantitatively comparing predictive models with the Partial Information Decomposition. 2019 Conference on Cognitive Computational Neuroscience. 2019. doi:10.32470/ccn.2019.1142-0
6. Jaworska K, Yi F, Ince RAA, van Rijsbergen NJ, Schyns PG, Rousselet GA. Healthy ageing delays the neural processing of face features relevant for behaviour by 40 ms. BioRxiv. 2019;3260092. doi:10.1101/326009
7. sNichols TE, Holmes AP. Nonparametric permutation tests for functional neuroimaging: a primer with examples. Hum Brain Mapp. 2002;15: 1–25. Available: https://www.ncbi.nlm.nih.gov/pubmed/11747097
