# Supplementary material for: Cortical tracking of speech in noise accounts for reading strategies in children
Source: PLoS Biol. 2020 Aug 26;18(8):e3000840. doi: 10.1371/journal.pbio.3000840 (PMC7478533; doi:10.1371/journal.pbio.3000840)
Supplement: S5 Methods — (DOCX) [file pbio.3000840.s005.docx]

# Supporting Information

## S5 Methods: Recording of video stimuli

The 12 video stimuli of narrators telling a story were recorded with a digital camera (Sony handycam, HDR-CX115E). Audio signals were recorded with both the internal microphone of the camera, and an independent high quality microphone (Sony linear PCM recorder, PCM-D50). Audio tracks were synchronized, and only the high-quality audio was kept. Video recordings were framed as head-shots, and recorded at 50 frames per second (videos were 1920 × 1080 pixels in size, 24 bits/pixel, with an auditory sampling rate of 44100 Hz). The camera was placed ~1 m away from the narrators, and the face spanned about half of the vertical field of view. Final images were resized to a resolution of 1152 × 864 pixels. A black old-style-TV-monitor frame was then added to the image.
