# Supplementary material for: Cortical tracking of speech in noise accounts for reading strategies in children
Source: PLoS Biol. 2020 Aug 26;18(8):e3000840. doi: 10.1371/journal.pbio.3000840 (PMC7478533; doi:10.1371/journal.pbio.3000840)
Supplement: S6 Methods — (DOCX) [file pbio.3000840.s006.docx]

# Supporting Information

## S6 Methods: Accuracy of speech envelope reconstruction

For each condition and participant, a global value of cortical tracking of the attended speech was evaluated for all left-hemisphere sensors at once, and for all right-hemisphere sensors at once. The decoder tested on a given condition was built based on MEG data from all the other conditions. This procedure was preferred over a more conventional cross-validation approach in which the decoder is trained and tested on separate chunks of data from the same condition because of the paucity of data (*i.e.*, at most ~2.4 min of data per condition). It is based on the rationale that the different conditions do modulate response amplitude but not its topography and temporal dynamics. In practice, electrophysiological data were band-pass filtered at 0.2–1.5 Hz (phrasal rate) or 2–8 Hz (syllabic rate), resampled to 10 Hz (phrasal) or 40 Hz (syllabic) and standardized. The decoder was built based on MEG data from –500 ms to 1000 ms (phrasal) or from 0 ms to 250 ms (syllabic) with respect to speech temporal envelope. Filtering and delay ranges were as in previous studies for phrasal [1,2] and syllabic CTS [3–6]. Regularization was applied to limit the norm of the derivative of the reconstructed speech temporal envelope [7], by estimating the decoder for a fixed set of ridge values (λ = 2^-10^, 2^-8^, 2^-6^, 2^-4^, 2^-2^, 2^0^). The regularization parameter was determined with a classical 10-fold cross-validation approach: the data is split into 10 segments of equal length, the decoder is estimated for 9 segments and tested on the remaining segment, and this procedure is repeated 10 times until all segments have served as test segment. The ridge value yielding the maximum mean RA is then retained. The ensuing decoder was then used to reconstruct speech temporal envelope in the left-out condition. RA was then estimated in 10 disjoint consecutive segments. We then retained the mean of this RA, leaving us with one value for all combinations of subjects, conditions, hemispheres, and frequencies of interest.

Significance of RA in each participant, condition, hemisphere and frequency range was assessed with a *t*-test on the RA values evaluated on 10 disjoint segments.

## References

1. Destoky F, Philippe M, Bertels J, Verhasselt M, Coquelet N, Vander Ghinst M, et al. Comparing the potential of MEG and EEG to uncover brain tracking of speech temporal envelope. Neuroimage. 2019;184: 201–213. doi:10.1016/j.neuroimage.2018.09.006
2. Bourguignon M, Baart M, Kapnoula EC, Molinaro N. Lip-reading enables the brain to synthesize auditory features of unknown silent speech. J Neurosci. 2019. doi:10.1523/JNEUROSCI.1101-19.2019
3. Ding N, Simon JZ. Emergence of neural encoding of auditory objects while listening to competing speakers. Proc Natl Acad Sci U S A. 2012;109: 11854–11859. doi:10.1073/pnas.1205381109
4. O’Sullivan JA, Power AJ, Mesgarani N, Rajaram S, Foxe JJ, Shinn-Cunningham BG, et al. Attentional Selection in a Cocktail Party Environment Can Be Decoded from Single-Trial EEG. Cereb Cortex. 2014;25: 1697–1706. doi:10.1093/cercor/bht355
5. Zion-Golumbic EM, Ding N, Bickel S, Lakatos P, Schevon CA, McKhann GM, et al. Mechanisms underlying selective neuronal tracking of attended speech at a “cocktail party.” Neuron. 2013;77: 980–991. doi:10.1016/j.neuron.2012.12.037
6. Lalor EC, Foxe JJ. Neural responses to uninterrupted natural speech can be extracted with precise temporal resolution. Eur J Neurosci. 2010;31: 189–193. doi:10.1111/j.1460-9568.2009.07055.x
7. Crosse MJ, Di Liberto GM, Bednar A, Lalor EC. The Multivariate Temporal Response Function (mTRF) Toolbox: A MATLAB Toolbox for Relating Neural Signals to Continuous Stimuli. Front Hum Neurosci. 2016;10: 604. doi:10.3389/fnhum.2016.00604
