# Supplementary material for: Cortical tracking of speech in noise accounts for reading strategies in children
Source: PLoS Biol. 2020 Aug 26;18(8):e3000840. doi: 10.1371/journal.pbio.3000840 (PMC7478533; doi:10.1371/journal.pbio.3000840)
Supplement: S1 Results — nCTS, normalized cortical tracking of speech. (DOCX) [file pbio.3000840.s007.docx]

# Supporting Information

## S1 Results: Contribution of visual cortical activity to nCTS

In lips conditions, wherein participants saw the narrator’s talking face, visual cortical activity driven by articulatory mouth movements could have contributed to nCTS values. This aspect is important for the interpretation of the relations involving the visual modulation in phrasal and syllabic nCTS. Therefore, we evaluated the contribution of visual cortical activity on nCTS estimation. For that, nCTS values were recomputed based on left- and right-hemisphere sensors, excluding a total of 32 sensors (out of 102) largely covering the occipital area. The difference between nCTS values obtained with all sensors and those obtained without visual sensors gave an estimation of the contribution of visual cortical activity to nCTS.

The contribution of visual cortical activity to nCTS was analyzed with linear mixed-effects modeling, exactly in the same way as done for nCTS values (in Results section “What is the nature of the information about reading abilities brought by measures of SiN processing and classical behavioral predictors of reading?”). This analysis revealed no statistically significant effect of the type of visual input, type of noise, hemisphere, or interaction thereof to the contribution of visual cortical activity to phrasal and syllabic nCTS (ps > 0.05).
