# Supplementary material for: Cortical tracking of speech in noise accounts for reading strategies in children
Source: PLoS Biol. 2020 Aug 26;18(8):e3000840. doi: 10.1371/journal.pbio.3000840 (PMC7478533; doi:10.1371/journal.pbio.3000840)
Supplement: S2 Results — nCTS, normalized cortical tracking of speech; RAN, rapid automatized naming. (DOCX) [file pbio.3000840.s008.docx]

# Supporting Information

## S2 Results: Side measures are redundant with RAN and digit span but not with modulations in phrasal nCTS

The information about reading brought by the 3 “side” measures (visual modulation in syllabic nCTS, phoneme suppression and phoneme fusion) was redundant with that brought by a subset (possibly all) of the 4 “main” measures (RAN, phonological memory, visual and informational modulation in phrasal nCTS). To identify this subset, we relied on the PID framework. In this analysis, PID assessed the nature of the information about reading abilities (target) brought by the 3 side measures (first set of explanatory variables) and each of the 4 main measures (second set of explanatory variables). The analysis was run separately for the modulation in syllabic nCTS, and for both measures of phonological awareness (phoneme suppression and phoneme fusion) at once. Overall, the results tended to show that the visual modulation in syllabic nCTS provided unique information about reading only with regard to the two phrasal nCTS modulations, and synergic information mainly with the two classical behavioral predictors of reading (see S5 Table). Similar results were obtained for the two measures of phonological awareness (see S6 Table).
