# Supplementary material for: Cortical tracking of speech in noise accounts for reading strategies in children
Source: PLoS Biol. 2020 Aug 26;18(8):e3000840. doi: 10.1371/journal.pbio.3000840 (PMC7478533; doi:10.1371/journal.pbio.3000840)
Supplement: S3 Results — (DOCX) [file pbio.3000840.s009.docx]

# Supporting Information

## S3 Results: Reading profile and reading deficit in the dyslexic group

Here, we better characterize the dyslexic group in terms of variability in reading profile and reading deficit.

First, we evaluated how many dyslexic readers showed a deficit in reading scores compared with controls in age. For that, reading scores of dyslexic readers were standardized according to those of controls in age as follows: The regression model to correct for age, time spent at school and IQ was estimated based on the reading scores of controls in age and then applied to the reading scores of the dyslexic readers and controls in age. Then, the mean and standard deviation of ensuing scores of controls in age were used to derive a reading z-score. Of the 26 dyslexic readers, 23 had a deficient score on at least one subtest (z-score below –1.5). In the remaining 3 dyslexic readers, the lowest z-score was below –1.4. Seventeen dyslexic readers had a deficient score on 3 or more subtests. Finally, 10 had a deficit in both irregular word and pseudoword reading, 2 in irregular word reading only, and 5 in pseudoword reading only. All this indicates that our dyslexic readers had a rather homogenous reading profile, characterized by similar reading difficulties in the two reading pathways.

Second, we used principal component analysis to characterize the spread in reading score across dyslexic readers and reading subtests (see S2 Figure). For that, reading scores of dyslexic readers were corrected for age, time spent at school and IQ and standardized according to their own distribution. The first principal component accounted for 77.7 % of the variance in reading scores. Its loading values were similar across reading subtests, meaning that it mainly captured variability in the degree of the reading deficit. The second principal component accounted for 14.9 % of the variance in reading scores. It can be interpreted as a contrast between reading accuracy and reading speed for real words (i.e., loading close to 0 for pseudowords). Other subtle distinctions between reading subtests accounted for 7.4 % of the variance in reading scores. For comparison purposes, the two first principal components of reading scores for controls in age identified similar patterns but left 12.0 % of the variance unexplained. In the same line, the variance of the reading strategy index was lower in dyslexic readers (𝜎2 = 0.48) than in controls in age (𝜎2 = 0.72), though not significantly so (F(25,25) = 0.66, p = 0.31). These results indicate that our dyslexic group was highly homogeneous in terms of reading profile, and at the very least, more homogenous than regular readers.
