# Supplementary material for: Cortical tracking of speech in noise accounts for reading strategies in children
Source: PLoS Biol. 2020 Aug 26;18(8):e3000840. doi: 10.1371/journal.pbio.3000840 (PMC7478533; doi:10.1371/journal.pbio.3000840)
Supplement: S4 Results — nCTS, normalized cortical tracking of speech. (DOCX) [file pbio.3000840.s010.docx]

# Supporting Information

## S4 Results: Are features of nCTS related to the importance of reading difficulties in dyslexia?

To determine whether and how the reading deficit in dyslexia relates to nCTS in noise, we identified with linear mixed-effects modeling 1) how nCTS in dyslexic readers is modulated by conditions and hemisphere and 2) the set of classical behavioral predictors of reading and features of nCTS in noise that bring significant information about reading abilities in dyslexic readers. These were the exact same analyses we conducted in regular readers, and here too, nCTS and behavioral scores of dyslexic readers were corrected for age, time spent at school and IQ.

S7 Table and S3 Figure show how nCTS in dyslexic readers varied in the different conditions and hemispheres. The pattern of variation of phrasal nCTS was similar to that seen in typical readers (see Table 2 and Figure 2). In contrast, syllabic nCTS was modulated only by the noise (and not by visual information and hemisphere). This suggests selecting the same features of phrasal nCTS for the modeling of reading abilities, and only the global level and informational modulation for syllabic nCTS. Still, for the sake of completeness and comparability with the analysis conducted in typical readers, we also introduced the visual and hemisphere modulation in syllabic nCTS.

S8 Table presents the final linear mixed-effects model fit to reading scores. It shows that RAN score and the global level of phrasal nCTS relate to global reading abilities. It also shows that the informational modulations in phrasal nCTS relate to reading abilities in a way that depends on the type of reading subtest. These effects are illustrated with simple Pearson correlations in S9 Table. As in typical readers, the time necessary to fulfil the RAN task was significantly negatively correlated with all reading scores. Surprisingly, the global level of phrasal nCTS correlated negatively with all reading scores, and the informational modulation in phrasal nCTS correlated (non-significantly) negatively with all scores indexing reading speed and (non-significantly) positively with the precision score. As a result, 1) the informational modulation in phrasal nCTS correlated positively with the difference between reading accuracy (Alouette accuracy score) and the mean of the 4 other scores indexing reading speed (r = 0.51; p = 0.0081), and 2) phrasal nCTS in babble noise was strongly negatively correlated with score indexing reading speed (see main text).
