# Supplementary material for: Cortical tracking of speech in noise accounts for reading strategies in children
Source: PLoS Biol. 2020 Aug 26;18(8):e3000840. doi: 10.1371/journal.pbio.3000840 (PMC7478533; doi:10.1371/journal.pbio.3000840)
Supplement: S1 Discussion — (DOCX) [file pbio.3000840.s011.docx]

# Supporting Information

## S1 Discussion: Supplementary Discussion

**Impact of the acoustic SNR**

We did not manipulate the SNR in SiN conditions in the present study. Instead, it was set to 3 dB so that the attended speech was always louder than the noise. Still, one could expect that large effect sizes would be uncovered in more challenging/discriminating listening conditions, as often encountered in classroom [1]. However, children’s SiN perception abilities are lower than adults’. Indeed, SiN perception abilities develop until late childhood (≥ 10 years) due to maturation of the auditory system and attentional abilities [2,3]. Moreover, our data showed that fewer subjects had significant CTS in babble than non-speech SiN conditions for phrasal CTS, and in all SiN conditions for syllabic CTS, with values in the most challenging SiN condition (same-gender babble noise) that were ~50% lower than those in the noiseless condition. Accordingly, setting speech SNR to 3 dB appeared to have made the task challenging enough for children, while ensuring that they could keep their attention focused throughout the experiment.

**Impact of the methods used to estimate CTS**

The amount of data per condition was limited to 2.5 min. Although it may seem little, we evidenced in a previous study that on average, ~30 s of MEG suffice to uncover significant CTS [4]. Moreover, CTS was significant, when assessed non-parametrically within-participant, in most of our participants in the least challenging conditions (phrasal, 100 %; syllabic, 94%). Of note, more data was used to estimate the regression model mapping MEG data onto reconstructed speech temporal envelope. Indeed, the model used to estimate CTS in each condition was trained on the ~20 min of data from all other conditions. This procedure substantially improved the estimation of CTS compared with a procedure wherein the model was trained and tested in a cross-validation scheme on the data from each condition separately (data not shown). Still, longer data acquisition could have produced more stable CTS estimates, and perhaps stronger associations with reading scores.

**Generalization to other languages**

Finally, it is important to remember that this study was conducted in French, a language with opaque alphabetic orthography, which has consequences on the development of the lexical and sublexical reading pathways (see [5] for a review). It would be crucial to generalize our results to other types of languages. Indeed, due to language specificities, it is most likely that the relation we uncovered in French between phrasal nCTS in babble noise and reading strategy may be weaker or stronger in other languages. For example, reading in non-orthographic languages needs to rely mostly on the lexical pathway. We therefore expect phrasal nCTS in babble noise to relate to global reading abilities in such a class of languages. On the other hand, in transparent languages, all words can be read through the non-lexical pathway. Because of that, alterations in lexical reading are less evident, and the relation between phrasal nCTS in babble noise and reading strategy may be weaker.

**CTS in noise in illiterate adults**

A better understanding of the impact of reading instruction on SiN perception and CTS in noise could be gained from future studies in illiterate adults. Since illiterate adults have poorer phonological awareness [6], observing altered tracking at syllabic rate (2–8 Hz) would strengthen the view that this aspect of CTS relates to phonological awareness. On the other hand, the relation between tracking at phrasal/sentential rate (0.2–1.5 Hz) and the degree of development of the mental lexicon might be more obvious in this population in which mental lexicon is typically less developed [7].

## References

1. Picard M, Bradley JS. Revisiting speech interference in classrooms. Audiology. 2001;40: 221–244. Available: https://www.ncbi.nlm.nih.gov/pubmed/11688542
2. Thompson EC, Woodruff Carr K, White-Schwoch T, Otto-Meyer S, Kraus N. Individual differences in speech-in-noise perception parallel neural speech processing and attention in preschoolers. Hear Res. 2017;344: 148–157. doi:10.1016/j.heares.2016.11.007
3. Sanes DH, Woolley SMN. A behavioral framework to guide research on central auditory development and plasticity. Neuron. 2011;72: 912–929. doi:10.1016/j.neuron.2011.12.005
4. Destoky F, Philippe M, Bertels J, Verhasselt M, Coquelet N, Vander Ghinst M, et al. Comparing the potential of MEG and EEG to uncover brain tracking of speech temporal envelope. Neuroimage. 2019;184: 201–213. doi:10.1016/j.neuroimage.2018.09.006
5. Ziegler J C, Goswami U. Reading Acquisition, Developmental Dyslexia, and Skilled Reading Across Languages: A Psycholinguistic Grain Size Theory. Psychological Bulletin. 2005;131: 3–29. doi.org/10.1037/0033-2909.131.1.3
6. Loureiro C de S, Braga LW, Souza L do N, Nunes Filho G, Queiroz E, Dellatolas G. Degree of illiteracy and phonological and metaphonological skills in unschooled adults. Brain Lang. 2004;89: 499–502. doi:10.1016/j.bandl.2003.12.008
7. Kosmidis MH, Tsapkini K, Folia V. Lexical Processing in Illiteracy: Effect of Literacy or Education? Cortex. 2006. 1021–1027. doi:10.1016/s0010-9452(08)70208-9
