# Supplementary material for: Cortical tracking of speech in noise accounts for reading strategies in children
Source: PLoS Biol. 2020 Aug 26;18(8):e3000840. doi: 10.1371/journal.pbio.3000840 (PMC7478533; doi:10.1371/journal.pbio.3000840)
Supplement: S1 Table — The two values provided for the noiseless condition correspond to two arbitrary subdivisions of the noiseless data to match the amount of data for the eight noise conditions. CTS, cortical tracking of speech. (DOCX) [file pbio.3000840.s012.docx]

# Supporting Information

## S1 Table

|  | Phrasal CTS | | | | | | | | | |
| --- | --- | --- | --- | --- | --- | --- | --- | --- | --- | --- |
|  | Noiseless | | Non-speech Noise | | | | Babble Noise | | | |
|  |  |  | Least-energetic | | Most-energetic | | Opposite gender | | Same gender | |
|  | Pics | | Pics | Lips | Pics | Lips | Pics | Lips | Pics | Lips |
| Left hemisphere | 100 | 100 | 100 | 100 | 100 | 100 | 91.8 | 97.3 | 90.4 | 97.3 |
| Right hemisphere | 100 | 100 | 100 | 100 | 100 | 100 | 94.5 | 98.6 | 90.4 | 95.9 |
| At least one hemisphere | 100 | 100 | 100 | 100 | 100 | 100 | 97.3 | 100 | 95.9 | 100 |
|  | Syllabic CTS | | | | | | | | | |
| Left hemisphere | 89 | 84.9 | 82.2 | 83.6 | 82.2 | 86.3 | 53.4 | 65.7 | 49.3 | 61.6 |
| Right hemisphere | 91.8 | 89 | 89 | 91.8 | 86.3 | 91.8 | 69.9 | 76.7 | 67.1 | 76.7 |
| At least one hemisphere | 97.3 | 94.5 | 94.5 | 94.5 | 87.7 | 94.5 | 78.1 | 86.3 | 76.7 | 79.4 |
