# Supplementary material for: Cortical tracking of speech in noise accounts for reading strategies in children
Source: PLoS Biol. 2020 Aug 26;18(8):e3000840. doi: 10.1371/journal.pbio.3000840 (PMC7478533; doi:10.1371/journal.pbio.3000840)
Supplement: S2 Table — Significant values (p < 0.05) are displayed in boldface, and marginally significant values are displayed in boldface and italicized. CTS, cortical tracking of speech. (DOCX) [file pbio.3000840.s013.docx]

# Supporting Information

## S2 Table

|  | redundant | | unique for phonological awareness | | unique for the feature of CTS in noise | | synergic | |
| --- | --- | --- | --- | --- | --- | --- | --- | --- |
|  | z | p | z | p | z | p | z | p |
| informational modulation in phrasal nCTS | -0.52 | 0.57 | **2.96** | **0.0060** | **3.34** | **0.013** | **1.06** | **0.097** |
| visual modulation in phrasal nCTS | 0.38 | 0.30 | **1.85** | **0.011** | **3.27** | **0.057** | 0.25 | 0.33 |
| visual modulation in syllabic nCTS | **4.75** | **0.0006** | **1.18** | **0.036** | 2.12 | 0.12 | **5.35** | **0.0010** |
