# Supplementary material for: Cortical tracking of speech in noise accounts for reading strategies in children
Source: PLoS Biol. 2020 Aug 26;18(8):e3000840. doi: 10.1371/journal.pbio.3000840 (PMC7478533; doi:10.1371/journal.pbio.3000840)
Supplement: S3 Table — nCTS, normalized cortical tracking of speech; RAN, rapid automatized naming. (DOCX) [file pbio.3000840.s014.docx]

# Supporting Information

## S3 Table

|  | redundant | | unique for visual modulation in syllabic nCTS | | unique for each of the main measures (listed on the left) | | synergic | |
| --- | --- | --- | --- | --- | --- | --- | --- | --- |
|  | z | p | z | p | z | p | z | p |
| informational modulation in phrasal nCTS | **3.34** | **0.0070** | **2.01** | **0.044** | **1.42** | **0.092** | **3.15** | **0.015** |
| visual modulation in phrasal nCTS | 1.22 | 0.13 | **1.66** | **0.067** | **2.16** | **0.037** | 0.96 | 0.15 |
| RAN | **4.85** | **0.0001** | **2.31** | **0.032** | 0.84 | 0.18 | **5.15** | **0.0018** |
| forward digit span | **6.13** | **<0.0001** | **2.19** | **0.034** | 0.50 | 0.25 | **7.54** | **0.0006** |
