# Supplementary material for: Cortical tracking of speech in noise accounts for reading strategies in children
Source: PLoS Biol. 2020 Aug 26;18(8):e3000840. doi: 10.1371/journal.pbio.3000840 (PMC7478533; doi:10.1371/journal.pbio.3000840)
Supplement: S4 Table — (DOCX) [file pbio.3000840.s015.docx]

# Supporting Information

## S4 Table

|  | redundant | | unique for visual modulation in syllabic nCTS | | unique for each of the main measures (listed on the left) | | synergic | |
| --- | --- | --- | --- | --- | --- | --- | --- | --- |
|  | z | p | z | p | z | p | z | p |
| informational modulation in phrasal nCTS | -0.25 | 0.50 | **3.08** | **0.013** | **1.74** | **0.061** | 0.58 | 0.21 |
| visual modulation in phrasal nCTS | **1.84** | **0.061** | **1.39** | **0.094** | 1.27 | 0.11 | 0.46 | 0.24 |
| RAN | **5.51** | **0.0002** | **2.14** | **0.038** | 0.40 | 0.30 | **4.45** | **0.0038** |
| forward digit span | **5.59** | **<0.0001** | **2.14** | **0.039** | 0.31 | 0.33 | **3.70** | **0.0073** |
