# Supplementary material for: Cortical tracking of speech in noise accounts for reading strategies in children
Source: PLoS Biol. 2020 Aug 26;18(8):e3000840. doi: 10.1371/journal.pbio.3000840 (PMC7478533; doi:10.1371/journal.pbio.3000840)
Supplement: S5 Table — CTS, cortical tracking of speech. (DOCX) [file pbio.3000840.s016.docx]

# Supporting Information

## S5 Table

|  | Phrasal CTS | | | | | | | | | |
| --- | --- | --- | --- | --- | --- | --- | --- | --- | --- | --- |
|  | Noiseless | | Non-speech Noise | | | | Babble Noise | | | |
|  |  |  | Least-energetic | | Most-energetic | | Least-energetic | | Most-energetic | |
|  | Pics | | Pics | Lips | Pics | Lips | Pics | Lips | Pics | Lips |
| Children with dyslexia | 100 | 100 | 100 | 100 | 100 | 100 | 100 | 100 | 100 | 100 |
| Controls in age | 100 | 100 | 100 | 100 | 100 | 100 | 100 | 100 | 100 | 100 |
| Controls in reading level | 100 | 100 | 100 | 100 | 100 | 100 | 100 | 100 | 96.2 | 100 |
|  | Syllabic CTS | | | | | | | | | |
| Children with dyslexia | 100 | 100 | 100 | 100 | 100 | 100 | 84.6 | 88.5 | 80.8 | 92.3 |
| Controls in age | 96.2 | 96.2 | 92.3 | 96.2 | 92.3 | 96.2 | 88.5 | 88.5 | 84.6 | 96.2 |
| Controls in reading level | 84.6 | 84.6 | 92.3 | 92.3 | 92.3 | 88.5 | 80.8 | 88.5 | 65.4 | 76.9 |
