# Supplementary material for: Cortical tracking of speech in noise accounts for reading strategies in children
Source: PLoS Biol. 2020 Aug 26;18(8):e3000840. doi: 10.1371/journal.pbio.3000840 (PMC7478533; doi:10.1371/journal.pbio.3000840)
Supplement: S6 Table — Factors are listed in their order of inclusion. nCTS, normalized cortical tracking of speech. (DOCX) [file pbio.3000840.s017.docx]

# Supporting Information

## S6 Table

|  | 𝒳2 | | p |
| --- | --- | --- | --- |
|  | df | value |  |
| phrasal nCTS |  |  |  |
| noise | 3 | 249 | < 0.0001 |
| visual | 1 | 92.9 | < 0.0001 |
| hemisphere | 1 | 6.52 | 0.011 |
| noise × visual | 3 | 56.3 | < 0.0001 |
| noise × hemisphere | 3 | 15.1 | 0.0017 |
| syllabic nCTS |  |  |  |
| noise | 3 | 173 | < 0.0001 |
