# Supplementary material for: Cortical tracking of speech in noise accounts for reading strategies in children
Source: PLoS Biol. 2020 Aug 26;18(8):e3000840. doi: 10.1371/journal.pbio.3000840 (PMC7478533; doi:10.1371/journal.pbio.3000840)
Supplement: S7 Table — Regressors are listed in their order of inclusion. (DOCX) [file pbio.3000840.s018.docx]

# Supporting Information

## S7 Table

|  | 𝒳2 | | p |
| --- | --- | --- | --- |
|  | df | value |  |
| RAN | 1 | 17.9 | < 0.0001 |
| global level of phrasal nCTS | 1 | 5.27 | 0.022 |
| informational modulation in phrasal nCTS dependant on reading score | 5 | 24.1 | 0.0002 |
