# Supplementary material for: Cortical tracking of speech in noise accounts for reading strategies in children
Source: PLoS Biol. 2020 Aug 26;18(8):e3000840. doi: 10.1371/journal.pbio.3000840 (PMC7478533; doi:10.1371/journal.pbio.3000840)
Supplement: S8 Table — ***p < 0.001, **p < 0.01, *p < 0.05, #p < 0.1. nCTS, normalized cortical tracking of speech. (DOCX) [file pbio.3000840.s019.docx]

# Supporting Information

## S8 Table

|  | RAN | global level of phrasal nCTS | Informational modulation in phrasal nCTS |
| --- | --- | --- | --- |
| Alouette accuracy | **-0.40*** | **-0.37#** | 0.22 |
| Alouette speed | **-0.65***** | **-0.49*** | **-0.37#** |
| Irregular words | **-0.70***** | **-0.49*** | -0.23 |
| Regular words | **-0.69***** | **-0.54**** | -0.24 |
| Pseudowords | **-0.65***** | **-0.48*** | -0.30 |
