# Supplementary material for: Cortical tracking of speech in noise accounts for reading strategies in children
Source: PLoS Biol. 2020 Aug 26;18(8):e3000840. doi: 10.1371/journal.pbio.3000840 (PMC7478533; doi:10.1371/journal.pbio.3000840)
Supplement: S9 Table — ***p < 0.001, **p < 0.01, *p < 0.05, #p < 0.1. nCTS, normalized cortical tracking of speech. (DOCX) [file pbio.3000840.s020.docx]

# Supporting Information

## S9 Table

|  | Phrasal nCTS in pics | Phrasal nCTS in lips | Syllabic nCTS in pics | Syllabic nCTS in lips | Visual modulation in syllabic nCTS |
| --- | --- | --- | --- | --- | --- |
| Alouette accuracy | –0.11 | –0.31 | –0.02 | 0.02 | 0.04 |
| Alouette speed | **–0.57**** | **–0.63***** | –0.26 | –0.07 | 0.19 |
| Irregular words | **–0.47*** | **–0.61***** | –0.34**#** | –0.22 | 0.10 |
| Regular words | **–0.56**** | **–0.63***** | –0.33**#** | –0.13 | 0.20 |
| Pseudowords | **–0.49*** | **–0.63***** | –0.19 | –0.13 | 0.04 |
| reading strategy index | –0.18 | –0.23 | –0.30 | –0.18 | 0.11 |
