# Supplementary figures and images for: Cortical tracking of speech in noise accounts for reading strategies in children
Source: PLoS Biol. 2020 Aug 26;18(8):e3000840. doi: 10.1371/journal.pbio.3000840 (PMC7478533; doi:10.1371/journal.pbio.3000840)

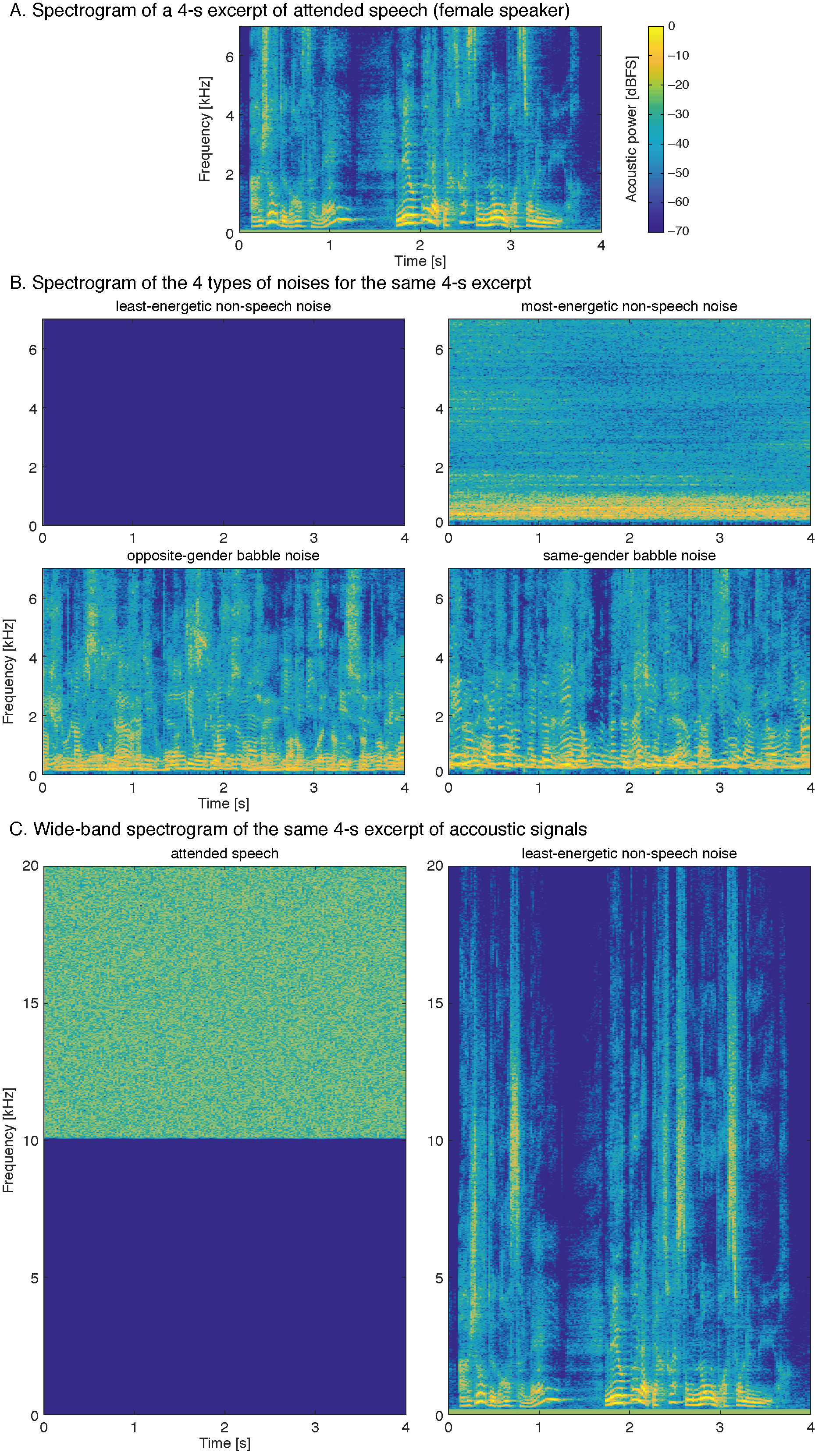

Supplement: S1 Fig — Spectrogram of a 4-s excerpt of attended speech (A) and corresponding noise (B) in the range of 0–7 kHz. Wide-band spectrograms (0–20 kHz) are also presented for the attended speech and the least-energetic nonspeech noise (C) to show that noise power was confined to frequencies above 10 kHz in this latter noise condition. The zeros of the dBFS were fixed based on the attended speech spectrogram and applied to all noise spectrograms. dBFS, decibel full scale. (TIF) [file pbio.3000840.s021.tif]

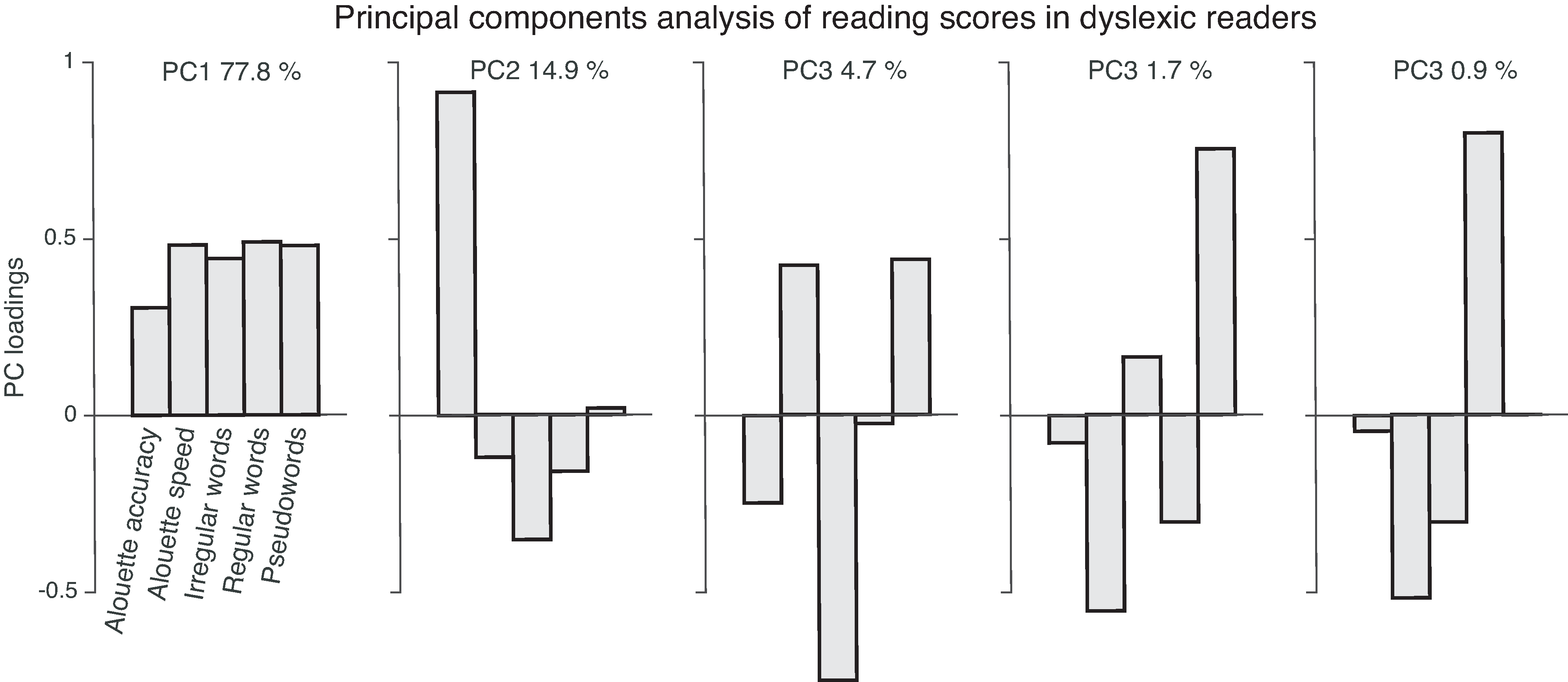

Supplement: S2 Fig — S6 Data contains the underlying data for this figure. nCTS, normalized cortical tracking of speech. (TIF) [file pbio.3000840.s022.tif]

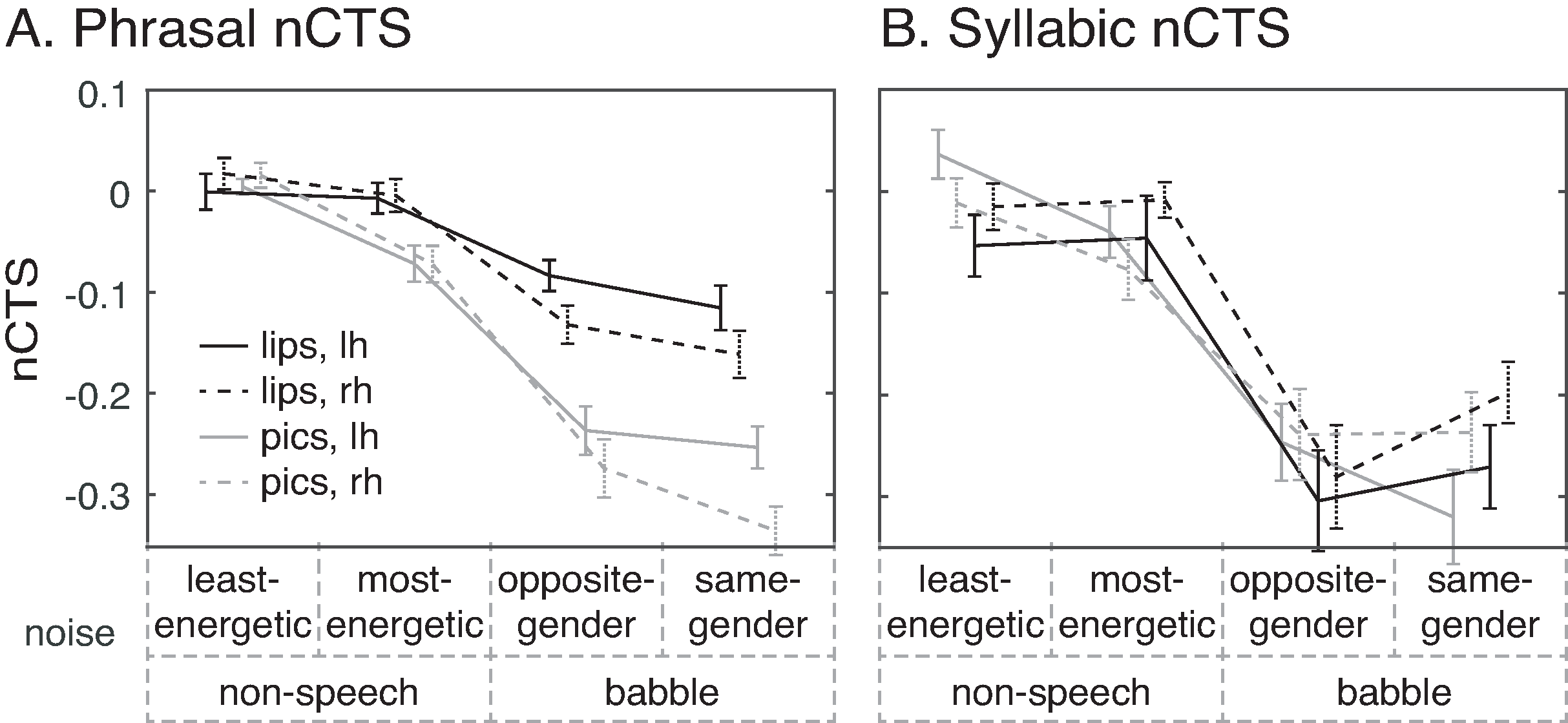

Supplement: S3 Fig — Impact of the main fixed effects on the nCTS at phrasal (A) and syllabic rates (B) in children with dyslexia. All is as in Fig 2. S7 Data contains the underlying data for this figure. nCTS, normalized cortical tracking of speech. (TIF) [file pbio.3000840.s023.tif]
