# Supplementary material for: Cortical tracking of speech in noise accounts for reading strategies in children
Source: PLoS Biol. 2020 Aug 26;18(8):e3000840. doi: 10.1371/journal.pbio.3000840 (PMC7478533; doi:10.1371/journal.pbio.3000840)
Supplement: Attachment — Submitted filename: SiN_tracking_and_reading_rebuttal.pdf [file pbio.3000840.s032.pdf]

Reviewer #1:

General comment

The manuscript presents an intriguing investigation of the relationship between indicators of reading skill and cortical tracking of speech in noise in the presence of additional visual (lip-movement, 'lips') information or in its absence ('pics'). They examine tracking at two time-scales, the phrasal and the syllabic rate. They compare the modulation of tracking by visual information in the presence of informational and non-informational noise.

As a result of the number of factors investigated and the number of outcomes presented, I found the paper overly dense and the conclusions hard to evaluate. Cortical tracking (nCTS) is evaluated for each hemisphere, for 2 visual conditions, for 4 noise conditions at 2 time-scales. These 32 values are then somehow reduced to 8 "contrasts" which are then related to a an array of reading indicators. This is achieved through a combination of variable selection using LME followed by PID.

The data point to a relationship between the enhancement of cortical tracking in noise by the provision of supporting visual information and several indicators of reading performance. The authors suggest that the ability to use visual information to enhance cortical entrainment to speech is related to reading ability. One potential mechanism offered is that in noisy classroom situations, children with better ability to track speech will benefit from easier development of the lexical route to reading for words with irregular orthographies.

The results are certainly of potential interest, but I have a number of concerns at the design level, about the way the data are interpreted and about the data presentation, which I found to be muddled. Two issues in particular stand out:

1) The conception of the different types of noise employed in the study
2) Frequent unguarded statements that imply direction of causation, that cannot be determined from the data, in part because of an absence of relevant behavioural data

I shall outline the concerns I have below.

General Answer

Comment 1

Fundamental Issues

I felt that there was at least one potentially fundamental flaw relating to the stimuli. The design of the experiment is articulated as a 2*2 parametric manipulation of informational * energetic masking. Unfortunately, it is simply not credible that masking can be non-energetic. Informational masking necessarily implies some degree of spectro-temporal coincidence of the target and the masker, in addition to the categorical coincidence (both are of the same nature as the target). A 'masker' that does not spectro-temporally overlap with a signal is not a masker, though it may have a deleterious impact on target processing for various reasons. The non-energetic non-informational condition is an interesting one, but since by design it aims to avoid masking the target it is difficult to see how these 4 'masking' conditions truly relate to one-another. It may be that the so-called non-energetic conditions are in fact distractors, but it is rather hard to determine what the conditions actually are based on the description in the methods in the main text. The supplementary materials only describe the generation of the energetic non-informational mask. More information is needed, and the terminology must be reconsidered. This has a considerable impact on the interpretation of the results. Furthermore, the difference between the masker types is not properly explored - to infer that it is the speech content of the informational conditions, and not their spectro-temporal modulation properties is not easily justified. This is, of course, a classical problem in the literature. How well are the non-informational and informational conditions really matched? Would a time-reversed version of the babble provoke the same kinds of effects, or not? It may be, of course that the authors do not want to draw specific conclusions based upon types of maskers. I would suggest

that an effort be made to ensure that the rationale behind the masker design and their relationship to the hypotheses be made clearer.

Answer 1

We thank the Reviewer for pointing at this important issue, and fully agree on the importance of better characterizing our stimuli. Accordingly, the spectral and spectrotemporal properties of the noise are now illustrated in Figure 1 & S1 (respectively). A full video stimulus can also be accessed at the following link:
https://drive.google.com/file/d/1Ap3Bb7iIsdoxDiQ3dLzJFwiu9CGtH9wc/view?usp=sharing
Ultimately, if the manuscript is accepted for publication, all stimuli will be deposited on OSF.

Also, we now report on "indices of energetic masking", obtained as described in the supplementary methods of the revised manuscript (line 15):

"Assessment of the degree of energetic masking

We characterized the degree of energetic masking introduced by the different types of noise, that is their degree of spectrotemporal overlap with the attended speech.

As a first step, we computed the spectrogram of the attended speech and that of the noise for each type of noise in each of the 4 versions of each of the 12 videos. Spectrograms were derived using 50-ms-long hanning windows sliding by steps of 25 ms, and with 5-Hz frequency resolution in the range 100–7000 Hz. The modulus of the Fourier coefficients were converted to a dB scale (log-transformed and multiplied by 20), which we denote $S_{attended}(t,f)$ for the attended speech and $S_{noise}(t,f)$ for the noise, with t indexing time and f indexing frequency. Figure S1 A & B presents the spectrogram of a 4-s excerpt of attended speech and all noises.

Indices of energetic masking ranging from 0 (no masking) to 1 (substantial masking) were derived from spectrograms. For that, we identified the indices of the 10% highest values of $S_{attended}(t,f)$, i.e., the set N so that $S_{attended}(t_n,f_n) > S_{attended}(t_x,f_x)$ for all indices $n \in N$ and $x \notin N$. For each index $n \in N$, we estimated the local energetic masking index as $M(n) = 1 - (S_{attended}(t_n,f_n) - S_{noise}(t_n,f_n))/(10\ dB)$ further thresholded between 0 and 1. Accordingly, $M(n) = 0$ when noise power ($S_{noise}(t_n,f_n)$) is at least 10 dB lower than attended speech power ($S_{noise}(t_n,f_n)$), $M(n) = 1$ when noise power exceeds attended speech power, and e.g., $M(n) = 0.6$ when noise power is 4 dB below speech power. The global energetic masking index was simply taken as the mean of $M(n)$ across all $n \in N$. The global energetic masking indices for each type of noise (12 videos times 4 versions) were averaged, and compared between types of noise with independent sample t-tests.

This showed that the global energetic masking was lowest for the least-energetic non-speech noise (0.00 ± 0.00; ps < 0.0001 for the comparison with the 3 other noises), highest for the most-energetic non-speech noise (0.428 ± 0.035; ps < 0.0001), and similar among opposite- and same-gender babble noises (0.310 ± 0.035 and 0.312 ± 0.035; $t(94) = 0.28$, $p = 0.78$)."

Based on this objective quantification of the degree of energetic masking, we have revised the naming of the different types of noises (see last paragraph of the text above). Few passages of the manuscript have been updated accordingly, including the following one in which we clearly state that, against our expectations, the two babble noises introduced a similar degree of energetic masking (line 135):

"*Each video featured 9 conditions: 1 noiseless and 8 SiN resulting from the combination of 4 types of noise with lips or pics visual inputs (Figure 1 & S1). The opposite- and same-gender babble noises introduced informational interferences, and a similar degree of energetic masking (see Supplementary Methods, subsection "Assessment of the degree of energetic masking"). The least- and most-energetic non-speech noises did not introduce informational interference, and a degree of energetic masking in accordance with their naming.*"

We expect time-reversed babble noise to introduce less interference since reversed speech is known not to introduce informational masking (Hoen et al. 2007, Rhebergen et al. 2005). In any case, following the principle that informational masking is conceptualized as anything that "reduces

intelligibility once energetic masking has been accounted for" (Cooke, Garcia Lecumberri, & Barker, 2008, p. 415), a babble noise does introduce some degree of informational masking (Hoen et al. 2007, Rhebergen et al. 2005). This information is now provided in the revised manuscript.
See Introduction, line 106:

"*The noise is informational when it is made up of other speech signals (as is the case of a multitalker babble, even in an unknown language, but not time-reversed), and non-informational otherwise [58–60].*"

It is also worth noting that we did not use a 2 x 2 design for the noise, but instead treated the types of noises as a single factor with 4 levels. This is now more obvious based on the change in nomenclature for the different types of noise.

Comment 2

A basic theoretical issue that I found somewhat disconcerting is the underlying assumption of the desirability of higher cortical tracking. It is as yet unproven whether more tracking somehow equates to better performance. Despite the community's best efforts, the causal relationship between speech tracking and speech comprehension is at best unclear.

Answer 2

We agree with the Reviewer that the functional role of CTS is still hotly debated. Still, an increasing body of research has related it to speech intelligibility. We now briefly mention recent evidence coming from transcranial alternating current stimulation.
See introduction section (page 5, line 91):

"*When listening to connected speech, human auditory cortical activity tracks the fluctuations of speech temporal envelope at frequencies matching the speech hierarchical linguistic structures, i.e., phrases/sentences (0.2–1.5 Hz) and words/syllables (2–8 Hz) [30–40]. Such cortical tracking of speech (CTS) is thought to be essential for speech comprehension [33,35,37,39,41–43]. Most convincingly, speech intelligibility can be enhanced by speech-matched transcranial electrical stimulation of auditory cortices [42,44].*"

Comment 3

Another serious concern is the complete absence of behavioural data on SiN. That is - without ascertaining that the differences in nCTS that are reported across groups and condition relate to SiN perception, claims cannot be made about the relationship between entrainment and SiN comprehension ability. This concern permeates the discussion. I do not question the various relationships that are reported between the nCTS and reading indicators but it does not seem tenable to make claims about how decreased AV fusion is responsible for impoverished SiN comprehension in dyslexic individuals. For instance: LL. 360-361 the measures are described as "objective cortical measure of the ability to deal with babble noise", this does not seem acceptable - where is the evidence that this reflects the ability to deal with babble noise? Do the participants report greater subjective clarity of the target, less effortful listening, higher accuracy of report? The one behavioural measure that seems to have been gathered is explicitly not analysed. I would strongly suggest that the authors reconsider this decision and attempt to link behaviour directly related to SiN to these measures in order to make their other conclusions more sustainable.

Answer 3

Following this comment, we have sought for associations between relevant features of nCTS and comprehension scores.
See Results section, line 347:

"***Is SiN comprehension accounted for by the features of nCTS related to reading?***

If the three features of nCTS related to reading abilities are to index relevant aspects of cortical SiN processing, we would expect them to directly relate to SiN comprehension. To

substantiate this consideration, we correlated these features of nCTS with a comprehension score computed as the percentage of correct answers to a total of 40 yes/no forced-choice questions. Again, all variables were corrected for age, time spent at school, and IQ. All 3 correlations were positive, but none of them were deemed significant (informational modulation in phrasal nCTS, $r = 0.16$, $p = 0.17$; visual modulation in phrasal nCTS, $r = 0.20$, $p = 0.082$; visual modulation in syllabic nCTS, $r = 0.09$, $p = 0.47$). The weakness of these associations could however be explained by ceiling effects in comprehension score due to comprehension questions being too simple. Indeed, 48% of the participants score 38/40 or more."

In fact, we used the same paradigm (but no assessment of reading abilities) in a study on a larger sample of participants ($n = 144$) spanning a larger age range. In this study, we will clearly demonstrate the presence of a weak (though this time highly significant) association between phrasal nCTS and speech comprehension ($r = 0.22$, $p = 0.0074$). We should be able to deposit this study on BioRxiv in the coming months, and refer to this study at a later stage of the revision.

Comment 4

A general question concerns the decisions to use the lexical/phonological route distinction and to take the existence of separate routes as a given. It may be the case that this applies to alphabetic languages, but it is well known that it cannot generalise to non-alphabetic languages (e.g. Chinese). Some effort should be made to acknowledge that this study focuses on an alphabetic language with a non-transparent orthography, which may represent a specific subcategory of how reading can be implemented.

Answer 4

We thank the Reviewer for bringing up these important distinctions between alphabetic vs. non-alphabetic orthographies, and transparent vs. opaque orthographies. These are now addressed in the revised manuscript.
See Introduction, line 79:

"*Following the Dual Route Cascaded (DRC) model, reading in languages with alphabetic orthographies is supported by two separate routes: the sublexical and the lexical routes [22,23].*"

See Discussion, line 610:

"***Further discussion***

In Supplementary Discussion, we discuss considerations related to that [...] the study was conducted in French (see Supplementary Discussion, subsection "Limitations and perspectives")."

See Supplementary Discussion, line 230:

"***Generalization to other language***

Finally, it is important to remember that this study was conducted in French, a language with opaque alphabetic orthography, which has consequences on the development of the lexical and sublexical reading pathways (see Ziegler and Goswami 2005 for a review). It would be crucial to generalize our results to other types of languages. Indeed, due to language specificities, it is most likely that the relation we uncovered in French between phrasal nCTS in babble noise and reading strategy may be weaker or stronger in other languages. For example, reading in non-orthographic languages needs to rely mostly on the lexical pathway. We therefore expect phrasal nCTS in babble noise to relate to global reading abilities in such a class of languages. On the other hand, in transparent languages, all words can be read through the non-lexical pathway. Because of that, alterations in lexical reading are less evident, and the relation between phrasal nCTS in babble noise and reading strategy may be weaker."

Comment 5

The authors further analyse the difference between a dyslexic group two non-dyslexic groups: one a group of reading-level matched children and a second group of age-matched children. The outcomes of this analysis are not straightforward, but they are summarised as follows; dyslexic individuals show the same "reliance on visual speech to boost phrasal nCTS" as age-matched controls, but phrasal nCTS in babble and reliance on visual speech to boost syllabic nCTS are altered. These results could be made substantially clearer and conclusions can only meaningfully be drawn from them if an explicit comparison between the reading-matched controls and the age-matched controls is carried out, to determine whether these effects are in any way specific to dyslexia.

Answer 5

Following this comment, we have worked on the presentation of the results in dyslexia (line 375):

"*Based on the result that reading abilities relate to phrasal nCTS in babble noise and to the boost in nCTS brought by visual speech, we focused the comparison on the phrasal nCTS in lips and pics averaged across hemispheres and babble noise conditions (see Fig. 4A). As a result, phrasal nCTS in pics was similar among individuals with dyslexia and controls in reading level, and higher in controls in age (significantly only for dyslexic readers; marginally for controls in reading level). In contrast, phrasal nCTS in lips was similar in all reading groups.*

Based on the result that reading abilities relate to the visual modulation in syllabic nCTS, we focused the comparison on this index (see Fig. 4B left part). This revealed that individuals with dyslexia had significantly lower visual modulation in syllabic nCTS than age-matched but not reading-level-matched controls; the two latter groups showing similar level of visual modulation in syllabic nCTS. To better understand the nature of this difference, we further compared between groups the syllabic nCTS in lips and pics averaged across hemispheres and noise conditions (see Fig. 4B right part). As a result, syllabic nCTS in pics was similar in all reading groups while in lips it was similar among individuals with dyslexia and controls in reading level, and higher in controls in age (significantly for dyslexic readers; marginally for controls in reading level)."

Comment 6

Major Issues

The repeated application of the PID methodology is challenging to follow. A little more effort to explain why each of the separate analyses is carried out, given that the method is presented as one that can provide insights into the unique contributions of a large set of variables. Naively, one would ask why all the variables of interest are not therefore handled together. Again, naively, one asks how legitimate it is to use LME to eliminate variables and then to use PID on selected variables only. To what extent could this be considered, in neuroimaging parlance, "double-dipping"? If the PID is intended only to provide qualitative insights then this is of no concern. It may be that it really is of no concern, and it would be welcome if the authors indicated this (and why).

Answer 6

We confirm to the Reviewer that there was no "double dipping" in our approach. This is because features of nCTS were selected based on the way they were modulated by conditions. They were not optimized to maximize correlation with any behavioral measure (including reading abilities). This is now briefly explained in the revised manuscript (line 202):

"*Note the absence of circularity in this approach since features of nCTS were not selected based on their relation with behavioral scores [68].*"

Feature extraction is a common procedure to limit the risk of introducing collinear regressors. This is true for the LME analysis, and also for PID analysis. In addition, feature extraction is an efficient means to improve the SNR on brain data. This is now briefly mentioned in the Results.

See line 203:

"And on a technical note, seeking association with a limited set of features of nCTS rather than with all nCTS values (32 = 4 noise conditions × 2 visual conditions × 2 hemispheres × 2 frequency ranges of interest) was necessary to avoid introducing close-to-collinear regressors in subsequent analyses, and to decrease random errors on nCTS estimates."

Comment 7

There is a consistent lack of clarity in what the measures are, and this should be rectified. For example, Table 3 refers to the following: "Regressors included in the final linear mixed-effects model fit to the 5 reading scores". This is presumably not the correct description, since the factors listed in the table are the 5 reading scores. What is the dependent variable in this analysis?

Answer 7

The dependent variables were the 5 reading scores (taken at once). This is now made clearer in the legend of Table 3 (see line 1284):

"Regressors included in the final linear mixed-effects model fit to the 5 reading scores (dependent variables)."

The legend of Table 2 has also been clarified in a similar way (see line 1278):

"Factors included in the final linear mixed-effects model fit to the normalized cortical tracking of speech (nCTS; independent variable) at phrasal rate and at syllabic rate. Factors are listed in their order of inclusion."

Comment 8

Methodological queries

A lot of relevant information is assumed or relegated to the supplementary materials. It would be helpful to make some of the more crucial aspects of the methodology (e.g. stimulus generation, interpretation of PID) more obvious.

Answer 8

The description of the generation of the most-energetic non-speech noise is now provided in the methods section (line 760):

"The most-energetic non-speech noise had its spectral properties dynamically adapted to mirror those of the narrator's voice ~1 s around. It was derived from the actual narrators' audio recording by i) Fourier transforming the sound in 2-s-long windows sliding by step of 0.5 s, ii) replacing the phase by random numbers, iii) inverse Fourier transforming the Fourier coefficients in each window, iv) multiplying these phase-shuffled sound segments by a sine window (i.e., half a sine cycle with 0 at edges, and 1 in the middle), and v) summing the contribution of each overlapping window."

Additional information has also been added on how the babble noises were build (line 770):

"For both babble noises, the 5 individual noise components were obtained from a French audiobook database (http://www.litteratureaudio.com), normalized, and mixed linearly."

In addition, two figures now illustrate the spectral and spectrotemporal properties of the different types of noise (line 758).

"Figures 1 and S1 illustrate their spectral and spectrotemporal properties."

Also see updated Figure 1 and new Figure S1.

To help readers understand PID analysis, we now refer to two successful applications of it in the main text (line 863):

"PID was previously used to decompose the information brought by acoustic and visual speech signals about brain oscillatory activity [80], and to compare auditory encoding models of MEG during speech processing [128]."

Comment 9
Numerous references are made to correcting variables for age, time in school, IQ, and then standardising (again). What was this correction? Would it not be possible to correct by the simple expedient of including the variables in the LME model?

Answer 9
We now give some more precisions about the nature of the correction, and provide a precise reference to the supplementary material (line 174).

"*In that analysis, nCTS values were corrected (linear regression intertwined with outlier fixing) for age, time spent at school and IQ (see Supplementary Methods, subsection "Preprocessing of brain and behavioral indices").*"

Introducing age in the LME is indeed a valid alternative strategy. However 2 facts led us not to opt for this apparently more desirable solution. (i) This approach is not compatible with correction for outliers. E.g., a reading score of 18 irregular words in 67 seconds is normal for a child aged 7, and an outlier for a child aged 10. (ii) The data needs to be corrected for age for the PID analysis.

Comment 10
Why did the model comparison procedure begin with the simplest rather than the maximal model?

Answer 10
It is our understanding that stepwise deletion and step up methods are common approaches to model selection in LME. We opted for the step up approach because of the exploratory nature of our analysis. But a stepwise deletion approach gave the same results. This is now mentioned in the revised manuscript.
See Methods (line 892):

"*Also worth noting, performing model selection with a stepwise deletion approach (i.e., when starting with the full model and iteratively removing fixed effects that did not decrease significantly model accuracy) yielded the exact same linear mixed-effects models.*"

See Supplementary Methods (line 106)

"*For cross-checking, some information values were also compared to their distribution obtained for explanatory variables permuted across subjects.*"

Comment 11
Why was LME used for variable selection and not stepwise regression?

Answer 11
We now provide a rationale for why we preferred LME over other statistical methods (see line 888):

"*Of note, we preferred linear mixed-effects modeling over other statistical methods for 2 reasons. (i) This method could identify both the factors that modulate nCTS and the regressors that explain reading scores. (ii) It could simultaneously model all the reading scores, and identify possible differences in correlation with the different readings scores.*"

Comment 12

The statistics reported for the various PID analysis need further elucidation. It is not clear what the statistic is, what the degrees of freedom are, nor how the p values are derived. Consequently the p values seem inconsistent, e.g. LL 207-208: "(redundant information =0.16; p = 0.0020; synergistic information = 0.12; p = 0.26)", but in LL.206 unique information = 0.31 corresponds to p=.10. How are we to interpret these figures? I accept that this information may be in the various existing publications on the PID, but it would be extremely helpful to be able to interpret these values in context without referring to these.

Answer 12

It is indeed not straightforward to interpret information figures, because they depend on the dimension of the sets of predictors and targets in a non-trivial way (i.e. the larger the sets, the larger the effect of limited sampling bias on the information measures). Following this comment, we decided to convert information measures into z-scores, which are more straightforward to interpret. This is now described in the Supplementary Methods, line 105:

"*We also computed the mean and standard deviation of the permutation distribution values to convert information measures into z-scores.*"

All the values in the text and table were corrected accordingly.

We also explain briefly in the revised methods that permutation statistics were used to assess statistical significance, and refer the readers to the Supplementary Methods for more details. See Results section, line 231:

"*For statistical assessment and conversion into easily interpretable z-scores, measures of information were compared to the distribution of these measures obtained after permuting reading scores across subjects (see Supplementary Methods, subsection "Partial information decomposition").*"

Given our usage of z-scores and non-parametric permutation statistics, it is not necessary to report on degrees of freedom.

Comment 13

It is not made clear why hemisphere is a variable of interest in the analyses - such a large-scale division of the brain seems somewhat arbitrary and should be clearly motivated.

Answer 13

We now provide a rationale for introducing the cerebral hemisphere as a factor in our analyses.

See Introduction, line 87:

"*Remarkably, the brain would implement these two reading strategies in two distinct neural pathways, mostly in the left hemisphere [25–29].*"

See Introduction, line 98:

"*In SiN conditions, children and adults' brain preferentially tracks the attended speech rather than the global auditory scene, though with reduced fidelity (especially reduced in the right hemisphere) when the noise hinders comprehension [30,31,40,46–56].*"

See Results, line 148:

"*CTS was computed as the correlation between the genuine and reconstructed speech temporal envelopes [...] for MEG sensor signals in the left and right hemispheres separately since the cortical bases of reading and SiN processing are hemispherically asymmetric [25–29,31,40].*"

Reviewer #2:

General comment

This manuscript presents research aimed at investigating the links between reading ability and 1) the cortical tracking of speech (as measured using MEG) and 2) classic behavioural predictors of reading in a population of schoolchildren. The authors present children with stories that either have no noise added or four different types of noise and that either are accompanied by a relevant static picture or a video of the speaker's face. They then calculate a measure of how well the MEG is tracking the speech in each of the 8 noise conditions normalized by the tracking in the no noise condition. The authors also collected a large number of measures of reading performance and a large number of measures of classical behavioural predictors. They then use linear mixed-effect modelling to explore how any of their 8 cortical tracking measures - together with their many classical behavioural predictors - might explain reading performance. Furthermore, they use Partial Information Decomposition to identify whether any of these predictors makes a unique contribution to predicting reading performance or whether it might be redundant with other predictors or whether it might combine with other predictors to make even better predictions (synergy). They find a number of relationships between cortical tracking measures and behavioural predictors. And they show that some of these relationships (but not others) apply to individuals with dyslexia.

This manuscript tackles an interesting topic and does so with a nice data set and nice experiment.

Comment 1

However, ultimately, I have one overarching concern that substantially dampens my enthusiasm for the work in its present form. Specifically, I could not help but worry about the robustness and replicability of the array of results we are presented with. The authors focus most of their analysis on 76 subjects. But they have 8 cortical tracking measures x 5 reading performance measures x 10 behavioral predictors (according to Table 1, but maybe only 5 in their analysis?). And, as such, I just found myself being sceptical about the results I was reading in every section. I would suggest that the authors might want to consider adding some additional analyses to reassure sceptical readers like me that the results we are seeing are likely to replicate. For example, the authors might consider permuting the labels on some of their predictors (e.g., the cortical tracking ones) and showing us that they can no longer get unique predictions from those cortical tracking measures. Or the authors might consider dividing their data in half and showing us that they consistently get the same pattern of results in both halves.

Answer 1

We agree with the Reviewer that the present study has to deal efficiently with the issue of multiple comparisons across the many brain and behavioral measures. This is actually the reason why we started with a single analysis (PID) to seek global associations between all different sets of variables. This point is now made clearer in the results section, before starting with the PID results (see line 213):

"*Having identified relevant features of cortical SiN processing, we first evaluated to which extent these features and classical behavioral predictors of reading bring information about reading abilities in a single, statistically controlled analysis.*"

We have also included the cross-check analysis proposed by the Reviewer, to ascertain that features of nCTS bring unique information about reading abilities, see line 240:

"*Further supporting the result that features of nCTS bring significant unique information about reading, this information measure was significantly higher than its permutation distribution where features of nCTS (rather than reading scores) were permuted across subjects ($p = 0.009$); and so was the value of redundant information ($p = 0.004$). Of notice, the unique information about reading brought by classical behavioral predictors was significantly higher when classical*

behavioral predictors were not permuted across subjects than when they were (p = 0.040); and so was the value of redundant information (p = 0.010)."

And in Supplementary material see line 106:

"*For cross-checking, some information values were also compared to their distribution obtained for explanatory variables permuted across subjects.*"

Finally, all behavioral measures were not expected to relate to reading abilities. This is why only 5 behavioral measures (called classical behavioral predictors of reading abilities) were used in the PID analysis. This is now better explained in the revised manuscript. See line 225:

"*The first set of explanatory variables, i.e, the classical behavioral predictors of reading, consisted of a total of 5 measures indexing phonological awareness (scores on phoneme suppression and fusion tasks), phonological memory (scores on forward and backward digit repetition), and RAN score.*"

Comment 2

I think the nCTS equation should be included in the main body of the text.

Answer 2

We now provide the equation of nCTS in the results section, when it is first introduced. See line 167:

"*The nCTS is a contrast between CTS in SiN (CTS_{SiN}) and noiseless ($CTS_{noiseless}$) conditions defined as*

$$nCTS = (CTS_{SiN} - CTS_{noiseless})/(CTS_{SiN} + CTS_{noiseless})$$

(see Methods for further technical details). It takes values between –1 and 1, with negative values indicating that the noise reduces CTS."

Comment 3

Sorry if I missed it, but I did not see the authors discuss the fact that cortical tracking of speech will be (uninterestingly) improved by the inclusion of a video of the speakers face because of the contribution of correlated activity from visual (i.e., occipital) sensors.

Answer 3

This is a very good point to raise. We now document the impact of visual activity on nCTS values. See Results, line 191:

"*Note that in lips conditions, wherein participants saw the narrator's talking face, visual cortical activity driven by articulatory mouth movements could have contributed to nCTS values. However, such visual contribution was actually negligible (see Supplementary Results, section "Contribution of visual cortical activity to nCTS").*"

And Supplementary Results, line 112:

"*Contribution of visual cortical activity to nCTS*

In lips conditions, wherein participants saw the narrator's talking face, visual cortical activity driven by articulatory mouth movements could have contributed to nCTS values. This aspect is important for the interpretation of the relations involving the visual modulation in phrasal and syllabic nCTS. Therefore, we evaluated the contribution of visual cortical activity on nCTS estimation. For that, nCTS values were recomputed based on left- and right-hemisphere sensors, excluding a total of 32 sensors (out of 102) largely covering the occipital area. The difference between nCTS values obtained with all sensors and those obtained without visual sensors gave an estimation of the contribution of visual cortical activity to nCTS.

The contribution of visual cortical activity to nCTS was analyzed with linear mixed-effects modeling, exactly in the same way as done for nCTS values (in Results section "What is the nature

of the information about reading abilities brought by measures of SiN processing and classical behavioral predictors of reading?"). This analysis revealed no statistically significant effect of the type of visual input, type of noise, hemisphere, or interaction thereof to the contribution of visual cortical activity to phrasal and syllabic nCTS (ps > 0.05)."

Comment 4

In line with my overarching concern above - I just found it implausible that nCTS could uniquely predict reading abilities when the classical behavioural predictors could not.

Answer 4

We were at first quite puzzled with this finding as well, but could make sense of it. We now explain better why it was so in the Results section (line 317):

"*This analysis identified an overall positive correlation between reading abilities and (i) the visual modulation in syllabic nCTS ($\mathcal{X}^2(1) = 9.74$, $p = 0.0018$), (ii) phoneme suppression ($\mathcal{X}^2(1) = 4.94$, $p = 0.026$) and (iii) phoneme fusion ($\mathcal{X}^2(1) = 4.00$, $p = 0.038$). Corresponding Pearson correlation coefficients are presented in Table 4. A detailed PID analysis revealed that these "side" measures were redundant—and synergistic to some extent—with RAN and forward digit span but not with visual and informational modulations in phrasal nCTS (see Supplementary Results, subsection "Side measures are redundant with RAN and digit span but not with modulations in phrasal nCTS"). Importantly, these results clarify why behavioral predictors of reading did not bring significant unique information about reading abilities: most of the variance in reading abilities they could explain (maximum |r| = 0.42; see Table 4) was also explained by the visual modulation in syllabic nCTS (maximum |r| = 0.37).*"

Comment 5

When you mention that "Two limitations are discussed in Supplementary Discussion", I think you should mention that they refer to limitations on only have one SNR for the stimuli and on training MEG models across all conditions and testing on each condition. Otherwise a reader is left wondering about/searching for those limitations.

Answer 5

We have followed this request. And because the revision process brought us to include more items in the Supplementary Discussion, we have now introduced a dedicated subheading.

See line 610:

"***Further discussion***

In Supplementary Discussion, we discuss considerations related to the fact that (i) only one acoustic SNR was studied, (ii) regression models to estimate CTS in a given condition were trained on all other conditions, (iii) occipital sensors were included in regression models to estimate CTS, and (iv) the study was conducted in French (see Supplementary Discussion, subsection "Limitations and perspectives")."

Comment 6

As I read the discussion - I could not help but wonder what the authors might expect to see in the cortical tracking of illiterate adults. Surely their cortex will reliably track speech in noise, no? Is there any literature to suggest that illiterate adults struggle more in challenging listening environments?

Answer 6

This is a very interesting question. To the best of our knowledge, the literature on SiN comprehension in illiterate individuals is non-existent. Some speculations can be made based on

studies showing that illiterate individuals have lower phonological awareness skills. Based on that, we would expect to observe a less accurate tracking of speech at syllabic rate (2–8 Hz) and normal tracking at phrasal/sentential rate (0.2–1.5 Hz). We now introduce this perspective in our Supplementary Discussion section to which we refer at the end of the Discussion

See Discussion, line 615:

"***Further discussion***

In Supplementary Discussion [...]. We also discuss the potential yield of future studies in illiterate adults."

See Supplementary Discussion, line 243:

"***CTS in noise in illiterate adults***

A better understanding of the impact of reading instruction on SiN perception and CTS in noise could be gained from future studies in illiterate adults. Since illiterate adults have poorer phonological awareness [137], observing altered tracking at syllabic rate (2–8 Hz) would strengthen the view that this aspect of CTS relates to phonological awareness. On the other hand, the relation between tracking at phrasal/sentential rate (0.2–1.5 Hz) and the degree of development of the mental lexicon might be more obvious in this population in which mental lexicon is typically less developed [138]."

Reviewer #3:

General comment

This study looked systematically at the association between cortical tracking of speech in noise and reading skill in children. The authors found that cortical tracking of the phrasal content of speech in noise is differentially related to lexical reading strategies as opposed to sublexical reading strategies. There was also evidence of differences in the cortical tracking of speech in noise of children with dyslexia, suggesting that they better integrate visual speech information to improve processing of phrasal level speech tracking, rather than syllable-level.

Major points:

This was a novel and interesting study with some clear findings and I appreciated the chance to review it. In the interest of transparency, while I have some expertise in neuroimaging, I do not have expertise in MEG specifically. However, I was able to follow the procedure and analysis and, to the best of my knowledge, the methodology appeared robust. There are some details that I am seeking clarification on in this review but, on the whole, it seems to me that enough detail is included to allow replication and scrutiny of methods. The sample size is good for a study of this nature. I have some concerns about how aspects of the data are interpreted but, in general, conclusions do not go too far beyond the findings and add value to the existing literature base in this area. The manuscript was very clear and well-written and it was a thought provoking study.

Comment 1

I have some concerns around the dyslexic sample, however, and I think that the manuscript needs to provide more detail about this subgroup and the analysis strategy taken. It is not clear anywhere that I could find how the dyslexic group were defined and recruited. Was it on the basis of existing diagnosis, or screening tests as part of the research project? How homogenous were the group in terms of their reading difficulties? This is particularly important because inferences are drawn in relation to reading strategies using findings from the dyslexic group. I'm also unclear why the authors choose not to look at the relationships between CTSiN and reading skill in the children with dyslexia. I appreciate that, due to statistical power issues, they may not be able to conduct the same analysis as for the control group. However, in order to support some of the key interpretations of what CTS deficits in the dyslexic group mean that are proposed in the discussion, some idea of whether the relationships (even in terms of basic correlations) look similar seems vital to me. It would be hard to argue that CTS deficits are of importance in the dyslexic profile if they don't seem to relate to reading skill in this group.

Answer 1

We now explain how participants were recruited. See line 640:

> "*Dyslexic readers had received a diagnosis of dyslexia, which implies that children had (at the time of diagnosis) at least 2 years of delay in reading acquisition that could not be explained by low IQ or social or sensitive disorders.*"

And see line 649:

> "*Participants were recruited mainly from local schools through flyer advertisements, or from social networks.*"

We demonstrate that our dyslexic group was rather homogenous in terms of reading profile. See Results section, line 400:

> "*In supplementary material we show that our dyslexic group was homogenous in terms of reading profile but not in the severity of the reading deficit (see Supplementary Results, subsection "Reading profile and reading deficit in the dyslexic group").*"

And see Supplementary Results, line 143:

"***Reading profile and reading deficit in the dyslexic group***

Here, we better characterize the dyslexic group in terms of variability in reading profile and reading deficit.

First, we evaluated how many dyslexic readers showed a deficit in reading scores compared with controls in age. For that, reading scores of dyslexic readers were standardized according to those of controls in age as follows: The regression model to correct for age, time spent at school and IQ was estimated based on the reading scores of controls in age and then applied to the reading scores of the dyslexic readers and controls in age. Then, the mean and standard deviation of ensuing scores of controls in age were used to derive a reading z-score. Of the 26 dyslexic readers, 23 had a deficient score on at least one subtest (z-score below –1.5). In the remaining 3 dyslexic readers, the lowest z-score was below –1.4. Seventeen dyslexic readers had a deficient score on 3 or more subtests. Finally, 10 had a deficit in both irregular word and pseudoword reading, 2 in irregular word reading only, and 5 in pseudoword reading only. All this indicates that our dyslexic readers had a rather homogenous reading profile, characterized by similar reading difficulties in the two reading pathways.

Second, we used principal component analysis to characterize the spread in reading score across dyslexic readers and reading subtests (see Supplementary Figure S2). For that, reading scores of dyslexic readers were corrected for age, time spent at school and IQ and standardized according to their own distribution. The first principal component accounted for 77.7 % of the variance in reading scores. Its loading values were similar across reading subtests, meaning that it mainly captured variability in the degree of the reading deficit. The second principal component accounted for 14.9 % of the variance in reading scores. It can be interpreted as a contrast between reading accuracy and reading speed for real words (i.e., loading close to 0 for pseudowords). Other subtle distinctions between reading subtests accounted for 7.4 % of the variance in reading scores. For comparison purposes, the two first principal components of reading scores for controls in age identified similar patterns but left 12.0 % of the variance unexplained. In the same line, the variance of the reading strategy index was lower in dyslexic readers ($\sigma^2 = 0.48$) than in controls in age ($\sigma^2 = 0.72$), though not significantly so ($F(25,25) = 0.66$, $p = 0.31$). These results indicate that our dyslexic group was highly homogeneous in terms of reading profile, and at the very least, more homogenous than regular readers."

Finally, we also investigate the relation between nCTS and reading abilities in dyslexic readers. See Results, line 400:

"*In supplementary material, we show that our dyslexic group was homogenous in terms of reading profile but not in the severity of the reading deficit (see Supplementary Results, subsection "Reading profile and reading deficit in the dyslexic group"). This raises the important question of whether and how the reading deficit in dyslexia relates to nCTS in noise. In supplementary material, we answer this question with the same linear mixed-effects modeling approach used in typical readers (see Supplementary Results, subsection "Are features of nCTS related to the importance of reading difficulties in dyslexia?"). However, the results are best illustrated by Pearson correlation between reading scores and nCTS in babble noise conditions in pics and lips (all measures corrected for age, time spent at school and IQ).*

Most surprisingly, phrasal nCTS both in lips and pics for dyslexic readers correlated significantly negatively with all reading scores indexing reading speed but not accuracy or strategy (see Fig. 5 and Supplementary Table S4). That is, the higher the phrasal nCTS, the slower they read. Beyond that, Supplementary Results (subsection "Are features of nCTS related to the importance of reading difficulties in dyslexia?") show that the informational modulation in phrasal nCTS correlated positively with the difference between reading accuracy and reading speed ($r = 0.51$; $p = 0.0081$). Syllabic nCTS in lips or pics for dyslexic readers did not correlate significantly with any of the reading scores (Supplementary Table S4)."

See Supplementary Results, line 175:

"*To determine whether and how the reading deficit in dyslexia relates to nCTS in noise, we identified with linear mixed-effects modeling 1) how nCTS in dyslexic readers is modulated by conditions and hemisphere and 2) the set of classical behavioral predictors of reading and features of nCTS in noise that bring significant information about reading abilities in dyslexic readers. These were the exact same analyses we conducted in regular readers, and here too, nCTS and behavioral scores of dyslexic readers were corrected for age, time spent at school and IQ.*

Supplementary Table S7 and Supplementary Figure S3 show how nCTS in dyslexic readers varied in the different conditions and hemispheres. The pattern of variation of phrasal nCTS was similar to that seen in typical readers (see Table 2 and Figure 2). In contrast, syllabic nCTS was modulated only by the noise (and not by visual information and hemisphere). This suggests selecting the same features of phrasal nCTS for the modeling of reading abilities, and only the global level and informational modulation for syllabic nCTS. Still, for the sake of completeness and comparability with the analysis conducted in typical readers, we also introduced the visual and hemisphere modulation in syllabic nCTS.

Table S8 presents the final linear mixed-effects model fit to reading scores. It shows that RAN score and the global level of phrasal nCTS relate to global reading abilities. It also shows that the informational modulations in phrasal nCTS relate to reading abilities in a way that depends on the type of reading subtest. These effects are illustrated with simple Pearson correlations in Supplementary Table S9. As in typical readers, the time necessary to fulfil the RAN task was significantly negatively correlated with all reading scores. Surprisingly, the global level of phrasal nCTS correlated negatively with all reading scores, and the informational modulation in phrasal nCTS correlated (non-significantly) negatively with all scores indexing reading speed and (non-significantly) positively with the precision score. As a result, 1) the informational modulation in phrasal nCTS correlated positively with the difference between reading accuracy (Alouette accuracy score) and the mean of the 4 other scores indexing reading speed ($r = 0.51$; $p = 0.0081$), and 2) phrasal nCTS in babble noise was strongly negatively correlated with score indexing reading speed (see main text)."

These new results are discussed as follows:
See abstract, line 47:

"*Finally, within dyslexic readers, measures of cortical representation of the phrasal content of SiN was negatively related to reading speed, and positively related to the compromise between reading precision and reading speed, potentially owing to compensatory attentional mechanisms.*"

See Discussion, line 498:

"*Our results in dyslexia support the existence of a relation between reading abilities and cortical measures of the ability to deal with speech in noise but bring important nuances. First, phrasal nCTS in non-visual babble noise conditions was altered in dyslexic readers compared with age-matched but not reading-level-matched controls, indicating that such alteration could be due to variability in reading experience. Second, within the dyslexic readers, phrasal nCTS was globally negatively correlated with reading speed, and the informational modulation in phrasal nCTS was positively correlated with the contrast between reading accuracy and reading speed. These two relations could be explained by compensatory attentional mechanisms, so that children with a severe dyslexia developed enhanced attentional abilities at the basis of improved SiN abilities and more accurate—despite still slower—reading (compared with children with a mild dyslexia). Hence, such relations might hold only in dyslexic readers free of attentional disorder as it was the case of our participants. Also, it should be reminded that these relations were found in a relatively small sample of dyslexic readers (n = 26), and should be confirmed by future studies.*"

And line 623:

"*However, within dyslexic readers, these relations appeared changed or even reversed, potentially owing to compensatory attentional mechanisms.*"

Comment 2
Similarly, can the authors provide details about the individual differences in CTS for the dyslexic group, as they do for the controls i.e. what percentage show statistically significant phrasal and syllable CTS? Important to know this is a reliable effect in that group in order to interpret their data.

Answer 2
This information is now provided in Supplementary Table S3.
We also mention the following in the Results section (line 365):
"*Supplementary Table S3 presents the percentage of the 26 children of each reading group (dyslexic readers, controls in age, and controls in reading level) showing statistically significant phrasal and syllabic CTS in each condition. All children showed significant phrasal CTS in all conditions except for one control in age that lacked significant CTS in one of the most challenging conditions (gender-matched babble noise without visual speech information). Qualitatively, fewer controls in reading level (than dyslexic readers and controls in age) showed significant syllabic CTS in all conditions. Still, the percentage of significant CTS remained above 80%, except for controls in reading level in the most challenging noise conditions (gender-matched babble noises), which indicates that CTS could be robustly assessed at the subject level in all reading groups.*"

Comment 3
A more minor point, but one that I think permeates several findings and discussions within the manuscript, is around the role of phonological awareness and how it has been tested. The relationships between reading and the phonological awareness measures are quite weak in this dataset. The authors rightly propose that this may be due to the age of the children and that phonological awareness becomes less central as reading becomes more automated. However, it is important for the authors to acknowledge the ceiling effects in their phonological awareness tests (~90% accuracy in control children, if I've interpreted tables correctly). It is much less likely that you will find phonological awareness mediates CTS effects if the tests are not sensitive enough, rather than because that skill does not mediate the relationship. I think that it is important that this is acknowledged as a possible reason why phonological awareness does not explain much of the variance in reading and why there may be no mediation effects. I think the conclusions relating to phonological processing need significant tempering because of this. In case of interest to the authors in their future work, we've found tests of spoonerisms to be more sensitive to phonological processing in these slightly older children who tend to perform towards ceiling on phoneme deletion or fusion tasks.

Answer 3
We thank the Reviewer for this very interesting comment. Indeed, we confirm there was a lack of sensitivity in our phonological awareness subtests. This might indeed explain the weak relation we uncovered between phonological awareness and reading abilities. We now raise this issue in the Discussion section.
See discussion section (line 602):
"*Nevertheless, the role of phonological awareness might have been underestimated in the present study, due to a lack of sensitivity in our phonological awareness subtests. Indeed, phonological awareness tasks revealed to be too easy for older participants, leading to ceiling effects (about half of the participants reached the maximum score on phoneme fusion and suppression tasks). This could explain the weak relation observed between reading abilities and phonological awareness skills. In contrast, there was no ceiling effect for the RAN, which may explain the strong correlation between this score and reading abilities.* »

Comment 4
Minor points
Line 41 - I think the authors should be cautious about claiming phrasal content of SiN relates to 'development of' lexical strategy when this is a concurrent association, not a longitudinal one. It is a little misleading.

Answer 4
It was indeed not our intention to make claims about causality. We now refer to the "*degree of development*" of the mental lexicon through the manuscript.

Comment 5
Line 201 - is PID analysis robust to the fact that one set of variables has 5 indicators and the other has 8? Seems like this could bias the analysis, but I'm not particularly familiar with this analysis approach, so would appreciate the authors' clarification on this.

Answer 5
This is an interesting question. When the number of samples (here participants) is very large compared with the number of regressors, differences in dimensionality between sets of regressors should not matter. In our case, obviously, the number of samples was rather restricted. Still, the same differences in dimensionality will be present in permuted data, so that our statistics takes this potential bias into account. This is more obvious in the revised manuscript as we now report z-scores rather than information values for PID measures (see answer to comment 12 of Reviewer #1).

Comment 6
Line 220 - what does 'and further standardised' mean?

Answer 6
The adverb "further" was here to be taken in the sense "in addition to what has already been done". In essence, it was dispensable, and we have removed it from the text.

Comment 7
Section starting with line 215 - Table of correlations show that visual modulation of syllable nCTS very consistently correlated with reading measures. Why doesn't this come out in the linear mixed-effects modelling? Is it because it doesn't contribute anything unique? It would be helpful for this to come through more clearly somewhere in this section.

Answer 7
Indeed, the visual modulation in syllabic nCTS was not retained in the final model because the variance in reading abilities it accounts for was already explained by the RAN and the forward digit span. This is now made clearer in the revised manuscript (line 317):

"*This analysis identified an overall positive correlation between reading abilities and (i) the visual modulation in syllabic nCTS ($\mathcal{X}^2(1)$ = 9.74, p = 0.0018), (ii) phoneme suppression ($\mathcal{X}^2(1)$ = 4.94, p = 0.026) and (iii) phoneme fusion ($\mathcal{X}^2(1)$ = 4.00, p = 0.038). Corresponding Pearson correlation coefficients are presented in Table 4. A detailed PID analysis revealed that these "side" measures were redundant—and synergistic to some extent—with RAN and forward digit span but not with visual and informational modulations in phrasal nCTS (see Supplementary Results, subsection "Side measures are redundant with RAN and digit span but not with modulations in phrasal nCTS"). Importantly, these results clarify why behavioral predictors of reading did not bring significant unique information about reading abilities: most of the variance in reading*

abilities they could explain (maximum |r| = 0.42; see Table 4) was also explained by the visual modulation in syllabic nCTS (maximum |r| = 0.37). And conversely, the visual modulation in syllabic nCTS was not retained in the final linear mixed-effects model of reading abilities for the same reason."

Comment 8
Lines 343-345 - The first and third relations referred to here seem to be to do with the link between CTS and reading skill so doesn't seem accurate to say that these were altered in the dyslexic group and relationships with reading skill weren't investigated in this group.

Answer 8
We have rectified the sentence as follows (line 431):

"*Finally, the features of nCTS underlying the first and third relations uncovered in typical readers (phrasal nCTS in babble noise, and visual modulation in syllabic nCTS) were significantly altered in dyslexia in comparison with aged-matched but not reading-level-matched typically developing children.*"

Other sentences that used the same wording have been corrected as well.
In the Introduction (line 121): "*Are these different aspects of cortical SiN processing altered in dyslexic children in comparison with typical readers matched for age or reading-level?*"

Comment 9
Lines 368-369 - I'm not clear how the results in dyslexia support this relation, particularly as children with dyslexia often have more significant difficulties with pseudoword reading than irregular word reading. Can this be clarified?

Answer 9
This issue is now clarified. See Results section, line 400:

"*In supplementary material, we show that our dyslexic group was homogenous in terms of reading profile but not in the severity of the reading deficit (See Supplementary Results, subsection "Reading profile and reading deficit in the dyslexic group").*"

And see Supplementary Results, line 154:

"*Finally, 10 [dyslexic readers] had a deficit in both irregular word and pseudoword reading, 2 in irregular word reading only, and 5 in pseudoword reading only.*"

And see Supplementary Results, line 168:

"*In the same line, the variance of the reading strategy index was lower in dyslexic readers (σ^2 = 0.48) than in controls in age (σ^2 = 0.72), though not significantly so (F(25,25) = 0.66, p = 0.31).*"

Comment 10
Line 518 - I think it's important to state the age range of the children somewhere here. I know it's in Table 1 but it's important information that needs to be found easily.

Answer 10
We now provide mean age, SD and range in the Methods section (line 637):

"*Seventy-three typical readers (mean ± SD age, 8.74 ± 1.41 years; age range, 6.70–11.72 years) and 26 dyslexic readers (mean ± SD age, 10.24 ± 1.08 years; age range, 7.97–12.29 years)*

enrolled in elementary school took part in this experiment (see Table 1 for participants' characteristics)."

Comment 11

Line 708 - A (very brief) description of what nCTS is would be beneficial here. I know it's described in the results but despite the ordering of the sections many people will read the method before the results.

Answer 11

We have added the requested description in the revised methods (line 840):

"*Based on CTS values, we derived the normalized CTS (nCTS) in SiN conditions as the following contrast between CTS in SiN (CTS_{SiN}) and noiseless ($CTS_{noiseless}$) conditions:*

$$nCTS = (CTS_{SiN} - CTS_{noiseless})/(CTS_{SiN} + CTS_{noiseless}).$$

Such contrast presents the advantage of being specific to SiN processing abilities by factoring out the global level of CTS in the noiseless condition. However, it can be misleading when derived from negative CTS values (which may happen since CTS is an unsquared correlation value). For this reason CTS values below a threshold of 10% of the mean CTS across all subjects, conditions and hemispheres were set to that threshold prior to nCTS computation. Thanks to this thresholding, the nCTS index takes values between –1 and 1, with negative values indicating that the noise reduces CTS."
