# Supplementary material for: Cortical tracking of speech in noise accounts for reading strategies in children
Source: PLoS Biol. 2020 Aug 26;18(8):e3000840. doi: 10.1371/journal.pbio.3000840 (PMC7478533; doi:10.1371/journal.pbio.3000840)
Supplement: Attachment — Submitted filename: SiN_tracking_and_reading_rebuttal_3.docx [file pbio.3000840.s033.docx]

REVIEWER'S COMMENTS:

**Reviewer #2:**

Many thanks to the reviewers for their efforts in addressing my previous comments. I think the manuscript is significantly improved.

One remaining comment: I still remain skeptical about the idea of using 8 different cortical tracking measures and 5 different classical behavioral predictors of reading to account for 5 measures of reading in 73 subjects. And saying that you do so "in a single, statistically controlled analysis" doesn't ease my skepticism I am afraid. So I will leave it as a suggestion to the authors that they might want to consider how they can try to make their approach more compelling to new readers. This could be, for example, by discussing the strengths and weakness of PID for detecting spurious vs real relationships. Or by including some other analysis that would convince a new reader that the results are likely to replicate and are not simply the result of overfitting to the present dataset.

**Answer to Reviewer #2:**

We understand the Reviewer’s concern, and still agree that it is extremely important to report robust results. However, we confirm that the PID analysis in combination with permutation statistics did exactly the following: identify the significance of an association between (i) reading abilities and (ii) behavioral and cortical SiN tracing measures, taking into account the dimensionality of the datasets. We now expand on this in the revised manuscript and hope this will alleviate the Reviewer’s skepticism. See S4 Methods:

“*The statistical significance of the different information values was assessed with a nonparametric permutation test [7]. A permutation distribution (based on 10,000 different random permutations) was computed for each information value by randomly permuting children’s target values (i.e., assigning target values of child 1, 2, 3, …, 73 to, e.g., child 34, 20, 61, …, 19). This permutation scheme specifically disrupts the associations between explanatory and target variables while preserving the associations within all explanatory variables and within all targets variables. For each information value, a significance level was computed as the proportion of values from the permutation distribution exceeding the observed value. We also computed the mean and standard deviation of the permutation distribution values to convert information measures into z-scores. Note that, owing to the generality of permutation statistics, significance levels are exact regardless of the strengths and weaknesses of the method used to estimate associations (here PID); and owing to the specificities of the permutation scheme we used, significance levels intrinsically take into account the dimensionality of the explanatory and target sets. Still, for cross-checking, some information values were also compared to their distribution obtained for explanatory variables permuted across subjects.*”
